# Differences in glutamate receptors and inflammatory cell numbers are associated with the resolution of pain in human rotator cuff tendinopathy

**DOI:** 10.1186/s13075-015-0691-5

**Published:** 2015-07-10

**Authors:** Benjamin John Floyd Dean, Sarah J. B. Snelling, Stephanie G. Dakin, Richard J. Murphy, Muhammad Kassim Javaid, Andrew Jonathan Carr

**Affiliations:** Nuffield Department of Orthopaedics, Rheumatology and Musculoskeletal Sciences, University of Oxford, Botnar Research Centre, Windmill Road, Oxford, OX3 7LD UK

## Abstract

**Introduction:**

The relationship between peripheral tissue characteristics and pain symptoms in soft tissue inflammation is poorly understood. The primary aim of this study was to determine immunohistochemical differences in tissue obtained from patients with persistent pain and patients who had become pain-free after surgical treatment for rotator cuff tendinopathy. The secondary aim was to investigate whether there would be differences in glutaminergic and inflammatory gene expression between disease-derived and healthy control cells in vitro.

**Methods:**

Supraspinatus tendon biopsies were obtained from nine patients with tendon pain before shoulder surgery and from nine further patients whose pain had resolved completely following shoulder surgery. Histological markers relating to the basic tendon characteristics, inflammation and glutaminergic signalling were quantified by immunohistochemical analysis. Gene expression of glutaminergic and inflammatory markers was determined in tenocyte explants derived from painful rotator cuff tendon tears in a separate cohort of patients and compared to that of explants from healthy control tendons. Dual labelling was performed to identify cell types expressing nociceptive neuromodulators.

**Results:**

Tendon samples from patients with persistent pain demonstrated increased levels of metabotropic glutamate receptor 2 (mGluR2), kainate receptor 1 (KA1), protein gene product 9.5 (PGP9.5), CD206 (macrophage marker) and CD45 (pan-leucocyte marker) versus pain-free controls (*p* <0.05). NMDAR1 co-localised with CD206-positive cells, whereas PGP9.5 and glutamate were predominantly expressed by resident tendon cells. These results were validated by in vitro increases in the expression of mGluR2, N-methyl-D-aspartate receptor (NMDAR1), KA1, CD45, CD206 and tumour necrosis factor alpha (TNF-α) genes (*p* <0.05) in disease-derived versus control cells.

**Conclusions:**

We conclude that differences in glutamate receptors and inflammatory cell numbers are associated with the resolution of shoulder pain in rotator cuff tendinopathy, and that disease-derived cells exhibit a distinctly different neuro-inflammatory gene expression profile to healthy control cells.

**Electronic supplementary material:**

The online version of this article (doi:10.1186/s13075-015-0691-5) contains supplementary material, which is available to authorized users.

## Introduction

Musculoskeletal pain is a common and costly healthcare problem affecting a third of the population. Shoulder pain is the third most common cause of musculoskeletal pain in the community and approximately 1 % of adults consult a general practitioner with new shoulder pain annually [1]. The majority of shoulder pain is associated with rotator cuff tendinopathy (RCT) [2, 3]. Tendon pain is more common in people with rotator cuff tears than those with normal tendons [4], however there is a poor association between the severity of symptoms and extent of structural change [5, 6] and current non-operative strategies are focused on steroid-based therapies with mixed results. There is a need to better understand the role of the peripheral tissue in tendinopathy-related pain to inform novel therapeutic options, possible pathways involve the glutaminergic and inflammatory systems [7–10].

Glutamate is an important amino acid involved in many key physiological processes including cell metabolism, pain sensitisation and collagen synthesis [11, 12]. Much of the literature has been limited due to the lack of availability of biologically representative control tissue. The inability to gather tendon-matched and age-matched control tissue from live donors has been a significant limiting factor in many previous studies [13]. The first aim of this study was to investigate whether there were histological differences relating to the glutaminergic and inflammatory systems between painful and pain-free human supraspinatus tendons; these tendons being appropriately matched in terms of both tendon macrostructure and patient demographics. We hypothesised that there would be significant neuro-inflammatory differences between these groups. The second aim was to investigate whether there would be differences in glutaminergic and inflammatory gene expression between rotator cuff tendon tear-derived and healthy control cells. We hypothesised that disease cells would have a significantly different gene expression profile compared to control cells.

## Methods

### Analysis of pathological human supraspinatus tissue

#### Tendinopathic cohorts: painful and pain-free groups

All patients were initially referred to a specialist upper limb clinic, after failing conservative management that included a minimum of one bursal steroid injection, and a course of physiotherapy or a home exercise programme. All patients were diagnosed with RCT by the clinical evaluation of a senior consultant shoulder surgeon. Patients were excluded if there was a full-thickness rotator cuff tear, any other significant shoulder pathology not involving the rotator cuff (osteoarthritis, frozen shoulder, instability, or previous fracture), more than three previous glucocorticoid injections, a glucocorticoid injection within 6 weeks of the treatment intervention and systemic steroid use. Structural integrity of the rotator cuff was assessed ultrasonographically in all patients and also by the operating surgeon at the time of surgery. All ultrasound scans were performed by an individual trained using a specific validated protocol that has been proven to be reliable [14].

Patients completed the Oxford Shoulder Score (OSS) [15], a well-validated and widely used clinical outcome measure. The OSS is a self-administered questionnaire validated for the study of shoulder pain. The OSS was originally designed for pre- and post-surgical outcomes and in terms of content, it has questions related both to pain and function. In terms of construct validity, it has been well validated against other scoring systems as a shoulder-specific questionnaire specifically for studying rotator cuff tears. For this study any non-perfect OSS was classified as symptomatic or ‘painful’ and perfect scores ‘pain-free’ or asymptomatic.

Supraspinatus tendon biopsies were taken from nine patients undergoing subacromial decompression (SAD) surgery (painful group) and from nine patients 5 years or more after undergoing SAD surgery in whom pain had resolved completely (pain-free group). Pain was judged to have completely resolved if a full OSS of 48 was documented; a full score is only possible if the patient reports no pain symptoms over the previous 4 weeks. Of note, all patients in the pain-free group had had significant pain before their surgery was performed as evidenced by a median OSS of 24 (range 20–40) pre-SAD. Ethical approval for this study was granted by the local research ethics committee (Oxfordshire REC B ref: 09/H0605/111 and Oxfordshire REC B ref: 12/SC/0028) and full informed consent (according to the Declaration of Helsinki) was obtained from all patients.

### Tendon biopsy technique

A percutaneous ultrasound-guided core biopsy technique was used to acquire the supraspinatus tendon tissue samples in the painful and pain-free patient groups. This validated technique is described in more detail elsewhere [16], and in brief, samples were collected just posterior to the leading edge of the supraspinatus tendon. This tendon sample is within 1 cm of the bony insertion of supraspinatus onto the greater tuberosity. One biopsy specimen was obtained per patient and each biopsy was approximately 2 mm by 2 mm by 5 mm in size. Biopsies were immediately placed into 10 % buffered formalin, subsequently wax embedded and sectioned (4 μm) using a microtome (Leica Microsystems Ltd, Milton Keynes, UK).

### Histology and immunohistochemical procedures

**Fig. 1:**
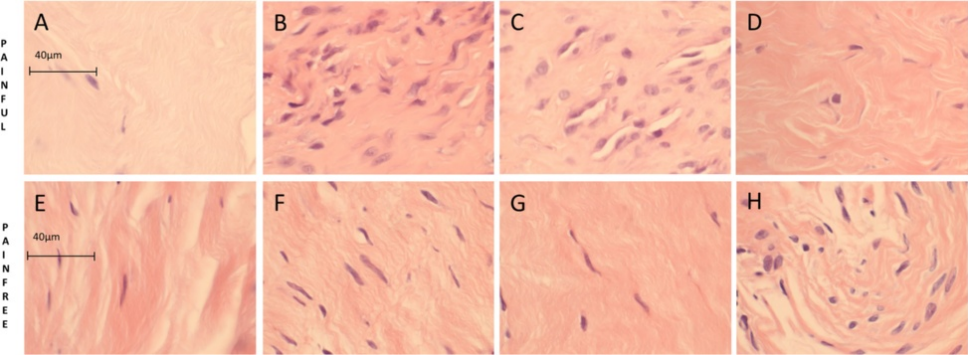
Basic histology was similar in the pain-free and painful groups. Photomicrographs show the haematoxylin and eosin staining in the rotator cuff tendon in the painful (**a**, **b**, **c**, **d**) and pain-free (**e**, **f**, **g**, **h**) groups. Scale bars: 40 μm

A haematoxylin and eosin (H&E) stain was performed on samples from every patient to assess the general characteristics of the tissue samples and to confirm that they were tendon (Fig. 1). Tissue sections were taken through deparaffinisation and target retrieval steps using an automated PT Link (Dako, Glostrup, Denmark). All antibody staining was performed using the EnVision FLEX™ visualisation system with an Autostainer Link 48 (Dako) according to the manufacturer’s instructions. Antibody binding was visualised using the FLEX™ 3,3’-diaminobenzidine (DAB) substrate working solution. Isotype control staining was also performed. To investigate the cell types expressing nociceptive neuromodulators in sections of pathological supraspinatus, dual-labelling immunofluorescence was performed with the macrophage marker CD206 and type B synoviocyte and nerve marker protein gene product 9.5 (PGP9.5), glutamate or N-methyl-D-aspartate receptor (NMDAR) 1. Details of antibodies used and their working concentrations, as well as all staining protocols can be found in the supplementary material (Appendix 1 in Additional file 1).

The assessment of the microscopic appearance of the slides immunostained with DAB was undertaken by a single blinded investigator. A Nikon inverted microscope (Nikon, Tokyo, Japan) using Axiovision software (Carl Zeiss, Jena, Germany) was used to capture images starting at one corner of the tissue moving systematically in a horizontal–vertical–horizontal manner until the tissue section was exhausted using ×100 magnification with oil immersion. A minimum of ten images were captured for each tissue section. Each antibody set was imaged using the same exact light intensity at the same sitting. The assessment of the microscopic appearance of the slides was undertaken using ImageJ® (National Institutes of Health, Bethesda, MD, USA) using validated methods to count cells and quantify staining, which have been described in detail elsewhere [8, 17]. Methods to quantify staining demonstrate good intra-observer (interclass correlation coefficient (ICC) = 0.830 at 1 month) and inter-observer reliability (ICC = 0.724–0.909) [17]. While cell-counting methods also demonstrated good intra-observer (ICC = 0.953 at 1 month) and inter-observer reliability (ICC = 0.970). Vascularity was measured using manual vessel counts of the CD34-stained sections (ICC = 0.921). Modified Bonar scores were also calculated using a scoring system based on the original scoring system and our group’s recent work [8, 18]; this is described in the online supplementary material (Appendix 2 in Additional file 1).

### Ex vivo analysis of disease-derived cells from a separate patient cohort and healthy control cells

#### Cell explant and analysis

Tendon tissue was obtained from the Oxford Musculoskeletal BioBank (Oxford REC C 09/H0606/11) and was collected with informed donor consent in full compliance with national and institutional ethical requirements, the United Kingdom Human Tissue Act, and the Declaration of Helsinki. Human tendon-derived cells were isolated by the explant culture of both rotator cuff tendon excised as part of debridement before tendon repair and hamstring tendon excess to requirements for autograft repair for anterior cruciate ligament rupture as previously described [19]. The rotator cuff tendon was taken from four male and three female patients with symptomatic rotator cuff tears (mean age 50 years +/− 11). The median duration of symptoms in the rotator cuff tear group was 12 months (range 6–24 months). The hamstring tendon was harvested from two healthy male and three female patients (mean age 27 years +/− 9). Tenocytes were cultured in Dulbecco’s modified Eagle’s medium (DMEM)-F12 containing 10 % fetal bovine serum (FBS) (Gibco, Life Technologies, Warrington, UK) at 37 °C and 5 % CO_2_. Cells were fed regularly every 2–3 days. RNA extraction was then carried out when passage 2 cells were at 80 % confluence 1 day after feeding.

### RNA extraction and cDNA synthesis

Cells were treated with TRIzol® Reagent (Life Technologies) and the samples were stored at −80 °C. RNA was then extracted using the Direct-zol™ RNA MiniPrep kit (Zymo Research, Irvine, CA, USA) according to the manufacturer’s instructions. This protocol included in-column DNase I digestion. The quality of the RNA was then tested using the NanoDrop 1000 spectrophotometer (Fisher Scientific, Loughborough, UK) prior to the samples being stored at −80 °C. The ratio of absorbance of 260 nm and 280 nm was used to determine the purity with all samples achieving a minimum of 1.80. Complementary DNA (cDNA) was synthesized using the transcriptor first-strand cDNA synthesis kit (Roche, Welwyn Garden City, UK) and was then stored at −20 °C until further use.

### Measurement of gene expression by quantitative RT-PCR

Real-time quantitative PCR were performed using the ViiA™ Real-Time PCR system (Fisher Scientific), a Fast SYBR® Green PCR kit (Life Technologies) and QuantiTect Primer Assays (Qiagen, Hilden, Germany), as well as validated assays designed by Primerdesign (Southampton, UK). Primer details and sequences are listed in the online supplementary material (Appendix 3 in Additional file 1). Samples without reverse transcriptase served as negative controls in real-time PCR to exclude genomic DNA contamination. No-template control was included to indicate there was no contamination in real-time PCR reagents. Samples were run in triplicate with a coefficient of variation between duplicates of <1.0 cycle, and the average value was used for analysis. A total of 250 ng of cDNA was used in each reaction whose volume was in total 20 μL. A total of 5 μL of cDNA was used within the total reaction volume of 20 μL. Standard curves were created for all genes measured and a set threshold of 0.15 was used for all calculations of relative gene expression. Relative quantification is expressed as 2^ΔCt^, where ΔCt is Ct (target gene) – Ct(TBP). Two housekeeping genes were used (TBP and β-actin) and results were consistent using both.

### Statistical analysis

Statistical analysis was carried using GraphPad Prism version 5.00 for Windows (GraphPad Software, San Diego, CA, USA). Histograms for all data sets were analysed. Data was normally distributed unless otherwise stated. Results are expressed as mean ± standard deviation unless otherwise stated. Unpaired *t* tests and Mann-Whitney *U* tests were used to test for differences between two groups for parametric and non-parametric data respectively. Statistical significance was set at a level of *p* <0.05. The Benjamini-Hochberg procedure was used to correct for multiple testing where stated. A power calculation (power = 0.8, α = 0.05, μ1 = 0.7, μ2 = 1.0, standard deviation 0.25, two-sided test) was used to determine that a sample size of n = 6 per group would be sufficient. Data from previous histological studies by our group was used to estimate the means and standard deviation used in this calculation. Spearman correlation coefficients were calculated to test the relationships between cell surface marker expression (CD206 for macrophages and PGP9.5 for type B synoviocytes), cytokine expression and glutamate receptor expression in the tendon tear-derived cells.

## Results

The age, gender and diseases characteristics of the painful and pain-free groups are shown in Table 1. The only significant demographic difference between the groups was presence of pain in the painful group and the absence of pain in the pain-free group as measured by the OSS (*p* = 0.0002). There were also no significant histological differences between groups in terms of cellularity, vascularity (vessel count) and proliferation (Table 2 and Fig. 1).

**Table 1:** Data representing the demographics of the pain-free and painful groups, quantification of the baseline questionnaires and tendon structure

	Pain-free group	Painful group	*p* value
Number	9	9	-
Age (mean)	52 (+/− 7.8)	51 (+/− 8.2)	0.72
Sex	6 M 3 F	7 M 2 F	1.0
Structure	7 intact + 2PTTs	7 intact + 2PTTs	1.0
Median length of symptoms pre-SAD (months)	22 (6–48)	12 (12–18)	0.43
Median number of steroid injections (range)	1 (1–3)	1 (1–4)	0.92
Oxford Shoulder Score (range)	48 (48)	32 (23–36)	0.0002^***^

All data is represented as median (interquartile range) or mean +/− standard deviation unless otherwise stated. Statistical significance is denoted by ^*^ *p* <0.05, ^**^ *p* <0.01, ^***^ *p* <0.001 (between groups). PTT denotes partial-thickness tear

**Table 2:** Data representing the basic histological characteristics and quantified immunohistochemistry

	Pain-free group	Painful group	*p* value
Mean vessel count	1.09 (+/− 0.63)	0.80 (+/− 0.65)	0.47
Mean nuclei count	11.6 (+/− 4.1)	8.0 (+/− 12.5)	0.57
Median modified Bonar score	7.0 (6.5–8.0)	8.0 (6.0–10.0)	0.59
Proliferation (% PCNA staining^a^)	0.04 (0.03–0.06)	0.04 (0.02–0.12)	0.86
HIF1alpha^a^	0.01 (0.01–0.04)	0.02 (0.01–0.04)	0.73
VEGF^a^	0.27 (+/− 0.18)	0.06 (+/− 0.05)	0.004^**b^
CD45 (% cells positive)	6.8 (4.7–10)	12.4 (8.1–16.8)	0.038^*^
CD206 (% cells positive)	13.2 (+/− 6.2)	26.8 (+/− 6.5)	0.0004^**b^
Lactate dehydrogenase^a^	0.001 (0.0004–0.002)	0.015 (0.01–0.04)	0.007^**b^
Glutamate^a^	0.003 (0.001–0.03)	0.004 (0.002–0.007)	0.86
NMDAR1^a^	0.013 (0.006–0.015)	0.025 (0.002–0.037)	0.61
PGP 9.5	0.87 (0.35–1.86)	3.75 (1.36–4.97)	0.0079^**b^
mGluR1^a^	0.43 (0.24–0.58)	0.36 (0.24–0.42)	0.55
mGluR2^a^	0.0019 (0.008–0.066)	0.064 (0.058–0.096)	0.05^*^
mGluR7^a^	0.18 (0.12–0.64)	0.005 (0.003–0.034)	0.0019^**b^
KA1	0.85 (1.31–2.62)	4.55 (2.37–7.15)	0.0028^**b^

All data is represented as median (interquartile range) or mean +/− standard deviation unless otherwise stated. Statistical significance is denoted by ^*^ *p* <0.05, ^**^ *p* <0.01, ^***^ *p* <0.001 (between groups).

*HIF1alpha* hypoxia-inducible factor 1-alpha*, KA1* kainate receptor 1, *mGluR* metabotropic glutamate receptor, *NMDAR1* N-methyl-D-aspartate receptor, *PCNA* proliferating cell nuclear antigen, *PGP 9.5* protein gene product 9.5, *VEGF* vascular endothelial growth factor

^a^Denotes per cell

^b^Denotes significant result after application of Benjamini-Hochberg procedure

### Neuro-inflammatory characteristics

**Fig. 2:**
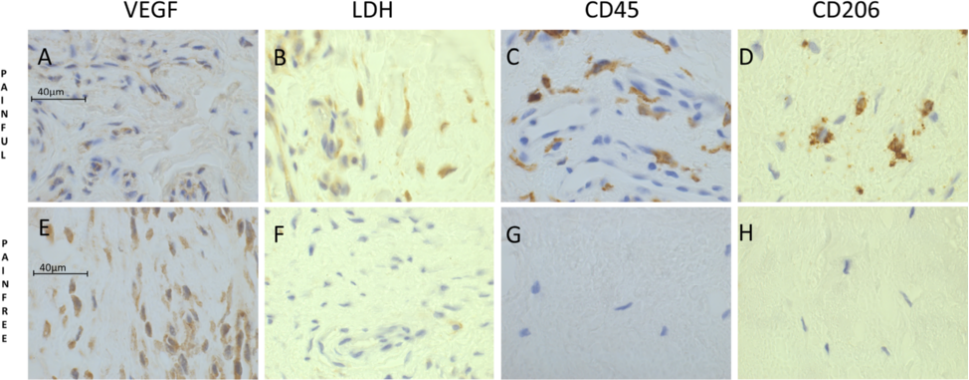
There were inflammatory differences between painful and pain-free rotator cuff tendons. Both the number of CD45 (pan-leucocyte marker) and CD206 (macrophage marker) positive cells were increased in the painful group versus the pain-free group. LDH expression was increased in the painful group, while VEGF was increased in the pain-free group. Photomicrographs depict the immunohistochemical staining of VEGF (**a**, **e**), LDH (**b**, **f**), CD45 (**c**, **g**), CD206 (**d**, **h**), in the painful and pain-free groups. Scale bars: 40 μm. *LDH* lactate dehydrogenase, *VEGF* vascular endothelial growth factor

Increased numbers of inflammatory cells were seen in the painful group versus the pain-free group (Table 2 and Fig. 2) as shown by the increased expression of the pan-leucocyte marker (CD45), a type B synoviocyte and nerve marker (PGP9.5) and a macrophage marker (CD206). CD45 and CD206 stained individual cells and did not stain whole vascular structures. NMDAR1 and PGP9.5 stained both individual cells and vascular structures. Of note, PGP9.5 is also a nerve marker but no distinct nerve-like structures could be reliably discriminated within the biopsy sections.

**Fig. 3:**
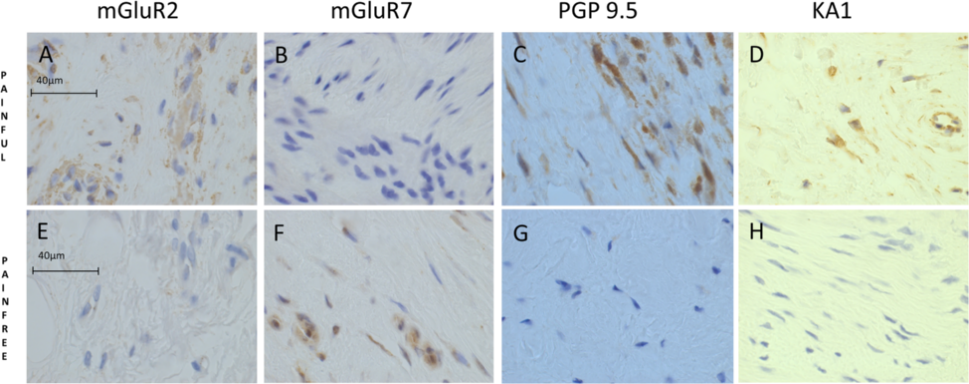
There were glutaminergic differences between painful and pain-free rotator cuff tendons. The expression of several glutaminergic markers was different between groups; mGluR2 and KA1 were increased in the painful group, while mGluR7 was increased in the pain-free group. mGluR7 staining was exclusively of endothelial cells, while mGluR2 and KA1 staining was of both endothelial cells and resident tendon cells. PGP 9.5 expression (type B synoviocyte marker) was increased in the painful group. Photomicrographs depict the immunohistochemical staining of mGluR2 (**a**, **e**), mGluR7 (**b**, **f**), PGP 9.5 (**c**, **g**) and KA1 (**d**, **h**). Scale bars: 40 μm. *KA1* kainate receptor 1, *mGluR* metabotropic glutamate receptor, *PGP9.5* protein gene product 9.5

**Fig. 4:**
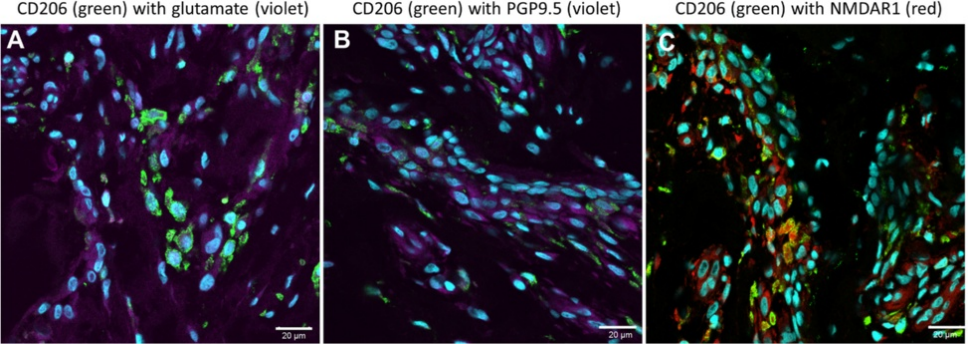
Representative immunofluorescent images of torn human supraspinatus to show cell types expressing nociceptive neuromodulators. Panel shows dual antibody labelling for CD206 (*green*) with (**a**) glutamate (violet), (**b**) PGP9.5 (*violet*) and (**c**) NMDAR1 (*red*). *Cyan* represents POPO-1 nuclear counterstain. NMDAR1 co-localised with CD206-positive cells, whereas PGP9.5 and glutamate were predominantly expressed by resident tendon cells. Scale bar: 20 μm. *mGluR* metabotropic glutamate receptor, *NMDAR* N-methyl-D-aspartate receptor, *PGP9.5* protein gene product 9.5

There were specific differences in the expression of glutaminergic markers between the painful and pain-free groups (Table 2 and Fig. 3); metabotropic glutamate receptor (mGluR)2 and kainate receptor 1 (KA1) were increased in the painful group, while mGluR7 was increased in the pain-free group. mGluR2 and KA1 staining related to individual cells as well as vascular structures. mGluR7 staining related solely to vascular structures. The KA1 and mGluR2 staining related to vascular structures as well as a sub-population of cells within the tendon. Dual-labelling immunofluorescence showed NMDAR1 co-localised with CD206-positive cells, whereas PGP9.5 and glutamate were predominately expressed by CD206-negative cells (resident tendon cells) (Fig. 4).

### Gene expression

**Fig. 5:**
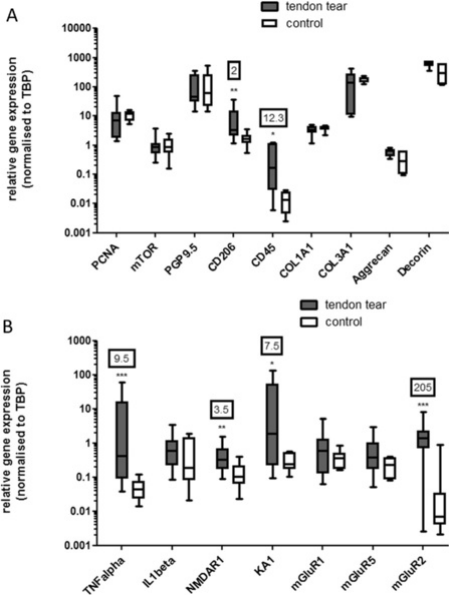
Neuro-inflammatory genes are upregulated in cells derived from rotator cuff tendon tears versus control cells. The expression of the TNF-α (9.5-fold, *p* = 0.0009), CD45 (12.3-fold, *p* = 0.03), CD206 (2-fold, *p* = 0.004), NMDAR1 (3.5-fold, *p* = 0.003), KA1 (7.5-fold, *p* = 0.049) and mGluR2 (205-fold, *p* = 0.0006) genes was significantly increased in tendon tear-derived cells (**a**) proliferation, cell surface markers and matrix genes (**b**) cytokine and glutaminergic genes (n = 7 tendon tear-derived and 5 controls). The *boxes* represent median +/− interquartile range, while the *whiskers* represent range. Numbers in *outlined boxes* represent fold change in gene expression. Statistical significance denoted by ^*^ *p* <0.05, ^**^ *p* <0.01, ^***^ *p* <0.001. *KA1* kainate receptor 1, *mGluR* metabotropic glutamate receptor, *NMDAR* N-methyl-D-aspartate receptor, *TNF-α* tumour necrosis factor alpha

The relative expression of the glutaminergic genes NMDAR1, KA1 and mGluR2 was significantly increased in cells derived from tendon tears compared to healthy control tendons (Fig. 5). mGluR7 gene expression was not detected in either disease or control cells. The gene expression of the metabotropic glutamate receptors mGluR1 and mGluR5 was not different between groups, although there was a trend towards increased expression of mGluR5 in the tendon tear-derived cells (*p* = 0.07). The relative expression of the inflammatory genes tumour necrosis factor alpha (TNF-α), CD45 and CD206 was significantly increased in tendon tear-derived cells versus control (Fig. 4), while interleukin-1β expression was not increased. There was no significant difference in extracellular matrix gene expression between groups (collagen type I alpha I chain (COL1A1), collagen type III alpha I chain (COL3A1), aggrecan and decorin). There was no difference in PGP9.5 (synoviocyte marker), PCNA (proliferating cell nuclear antigen) and mTOR (mammalian target of rabamycin) gene expression between groups.

### Correlation coefficients of gene expression data for tendon tear-derived cells

Within the gene expression data related to cells derived from rotator cuff tears there were strong correlations between CD206 expression and glutamate receptor expression; KA1 (*r* = 0.96, *p* <0.0001), NMDAR1 (*r* = 0.76, *p* = 0.002), mGluR1 (*r* = 0.83, *p* = 0.0002), mGluR2 (*r* = 0.95, *p* = 0.0002) and mGluR5 (*r* = 0.95, *p* = <0.0001). There was no strong or significant correlation (*r* >0.4) between PGP9.5 expression and glutamate receptor expression (*r* = 0.003, *p* = 0.86 for NMDAR1; *r* = 0.16, *p* = 0.14 for KA1; *r* = 0.07, *p* = 0.38 for mGluR2). There was a strong correlation between CD206 expression and TNF-α expression (*r* = 0.97, *p* <0.0001), while there was no strong or significant correlation between PGP9.5 expression and TNF-α expression.

## Discussion

Both the in vivo and ex vivo components of this work show that the glutaminergic and inflammatory pathways are active in painful tendinopathy, despite there being no significant differences in basic tendon histology between painful and pain-free tendons. The close relationship between the glutaminergic and inflammatory systems was further supported by the co-localisation of NMDAR1 and CD206, as well as the significant correlations between glutamate receptor and inflammatory gene expression.

There is little evidence in the literature relating histological changes in tissue to symptoms [20]. Tomita et al. [21] showed that an increased neural density in the subacromial bursa correlated with the presence of shoulder pain at rest. Gotoh et al. [22] demonstrated that substance P levels in the subacromial bursa appeared to correlate with pain levels as measured with a visual analogue score. A significant limitation with these two studies is the differences in gross structure that may explain the observed relationships with pain. The same limitation is relevant to much work carried out on other tendinopathies such as the Achilles and patellar, with controls invariably having normal gross tendon structure as opposed to the abnormal gross tendon structure frequently seen in disease [20].

The role of inflammation in tendinopathy has been the subject of significant controversy [7]. Some studies have demonstrated that greater numbers of inflammatory cells are present in tendinopathic than in healthy control tissues [23, 24], while recent evidence has related these inflammatory changes to patient symptomatology in the Achilles [25]. Increased levels of inflammatory cytokines such as interleukin-1β have repeatedly been demonstrated in tendinopathy. We demonstrate increased numbers of CD45 (pan-leucocyte) and CD206 (macrophage) positive cells in painful versus pain-free tendons. Pro-inflammatory markers TNF-α and CD45 were upregulated in disease cells versus controls; therefore the findings of the present study support the concept that inflammation contributes to the pain symptomatology in tendinopathy.

The exact phenotype of the tendon-derived cells is unclear, but the pattern of gene expression (CD45/CD206/TNF-α/PGP9.5) is consistent with the presence of a significant proportion of ‘synoviocyte’-type cells. Type A synoviocytes are resident macrophages and are also referred to as ‘fibrocytes’ [26, 27], they arise from monocyte precursors and are consequently CD45 positive [28]. It is likely that type A synoviocytes make up a proportion of the CD45- and CD206-positive cells seen in the in vivo study but it is also possible that a significant proportion of these cells migrate into tendon via the bloodstream. The increase in PGP9.5 in the painful group may relate to a subpopulation of type B synoviocytes (‘fibroblast-like synoviocytes’) as PGP9.5 has been described as a marker of this population of cells in animals [29]. The cell markers expressed by ‘synoviocytes’ and other tissue fibroblasts are extremely fluid and complex [30]. Emerging evidence points to the potential role of the ‘synoviocyte’ in several chronic inflammatory disorders [28] and this is supported by our findings. The level of PGP9.5 gene expression was 14-fold higher than CD206 expression in the tendon tear-derived tendon cells, demonstrating that type B synoviocytes are likely to be more abundant in vitro than the type A macrophage-like cells.

Glutamate receptors can be broadly broken down into two major types: ionotropic, which are glutamate-gated ion channels (iGlu), and metabotropic, which are G-protein coupled receptors that modulate signal transduction cascades (mGlu) [31]. The ionotropic receptors include kainate (KA) receptors, α-amino-3-hydroxy-5-methyl-4-isoxazole propionic acid (AMPA) receptors and N-methyl-D-aspartate (NMDA) receptors [32]. Glutaminergic changes have been described in painful tendinopathy, including an increase in extracellular glutamate concentration and the upregulation of NMDA receptors [8, 33, 34]. In the current study, the in vivo glutaminergic changes involving the mGluR2, mGluR7 and KA1 receptors alongside the metabolic change related to lactate dehydrogenase (LDH) are of interest; of note, both mGluR2 and KA1 gene expression were also upregulated in disease-derived cells in vitro alongside NMDAR1. Interestingly mGluR2 has been shown to be an endogenous inhibitor of inflammatory pain in mice [35], while peripheral mGluR2 agonism has analgesic properties in rats [36].

The co-localisation of NMDAR1 and CD206 suggests that the non-neurovascular glutamate receptors are predominantly located on the type A synoviocyte cell population; while the absence of NMDAR1 and PGP9.5 co-localisation suggests that this is not the case for the type B synoviocytes. This is further supported by the correlations between the glutamate receptor expression (KA1, NMDAR1, mGluR1, mGluR2 and mGluR5) and CD206 expression, alongside the lack of correlation between PGP9.5 expression and glutamate receptor expression. Therefore, it is highly likely that glutaminergic signalling machinery is predominantly expressed by the macrophage-like type A synoviocytes in vitro rather than the type B cells. The strong correlation between TNF-α gene expression and CD206 infers that the TNF-α secreted in disease is largely produced by the type A synoviocytes although further work is required to support this observation. PGP9.5 has been shown to co-localise with NMDAR1 on nerves in tendinopathy [34]. Unfortunately it was not possible to confirm this finding due to the paucity of nerves within the biopsy sections.

It should be noted that the staining relating to both KA1 and mGluR7 was to a large degree of vascular structures. This is the likely explanation for the absence of mGluR7 expression in explanted cells (fibroblasts likely massively outnumber endothelial cells in vitro). The decreased mGluR7 staining previously demonstrated in tendon tears versus healthy control is also largely related to endothelial cells and not the tendon cells [8]. In this study, the result does not appear to be as a result of the potential confounding factor vascularity, as this was not significantly different between the groups. Little is known about the role of mGluR7 in endothelial cell function but mGluR7 is known to be expressed by rat endothelial cells [37], while NMDA receptors are expressed by human endothelial cells [38]. NMDAR activity is decreased by mGluR7, inferring that the greater expression of mGluR7 in pain-free tendons may be representative of a less stressful environment for the endothelial cells given NMDAR’s potential pro-survival role [39]. The significant differences in endothelial cell staining (mGluR7 and KA1) seen does suggest that future research to investigate the role of endothelial cells in tendinopathy may have great potential in terms of better understanding disease pathogenesis. The finding that ionotropic glutamate receptor agonism increases inflammatory cytokine production [40–42] in human synoviocytes and chondrocytes suggests that glutamate receptor modulation may be a viable means of therapeutically targeting the synoviocyte in painful tendinopathy; this is consistent with our study’s finding in terms of the co-localisation of NMDAR1 and CD206. The kainate receptor has been implicated in the pathogenesis of osteoarthritis and KAR antagonism has shown therapeutic in slowing down disease progression in animal models [43]. It is worth adding that although this study focused primarily on the glutaminergic system, the signalling systems relating to other neurotransmitters such as acetylcholine and noradrenaline remain justifiable targets for further investigation [44, 45].

A strength of this study is that the tendon samples used for histological analyses were well matched in terms of patient demographics and tendon macrostructure. Only a minority of studies have used supraspinatus as a control and of these studies the vast majority have used cadaveric supraspinatus tendon. Only four studies have used supraspinatus from a live donor as a control [46–49]; three of these used the supraspinatus of patients who had sustained significant shoulder trauma and one used supraspinatus from patients undergoing surgery for shoulder instability [13]. To our knowledge this is the first histological study that has used structurally and age-matched tendon from pain-free tendinopathic patients as a control. The histological findings of the study in terms of protein expression have also been validated by the gene expression results. A limitation of this study is that the control cells used in the cell culture studies were not age-matched with the disease cells and were from a functionally different tendon, hamstring. The observational nature of our work means that any mechanisms underlying the causality of changes observed can only be inferred and not robustly proven. The cause of these tendon and cellular differences cannot be determined given the study’s cross-sectional design. The pain-free group had all had previous subacromial decompression surgery prior to becoming pain-free. This means that the tendon differences between groups may be as a result of a true surgical treatment effect or the natural history of the disease. The mechanism of the treatment effect after subacromial decompression surgery is unclear [50, 51].

## Conclusions

This study has shown that differences in specific glutamate receptors (KA1/mGluR2/mGluR7) and inflammatory cell numbers (CD45/CD206) are associated with the resolution of pain in rotator cuff tendinopathy. These findings improve our understanding of pain in tendinopathy and may help identify novel therapeutic targets.

## Additional file

Additional file 1:
**‘Appendices’ and consists of Appendix 1, 2 and 3.**

## Abbreviations

cDNA: complementary DNA
COL1A1: collagen type I alpha I chain
COL3A1: collagen type III alpha I chain
DAB: 3,3’-diaminobenzidine
ICC: interclass correlation coefficient
KA1: kainate receptor 1
mGluR: metabotropic glutamate receptor
NMDA: N-methyl-D-aspartate
NMDAR: N-methyl-D-aspartate receptor
OSS: Oxford Shoulder Score
PGP9.5: protein gene product 9.5
PTT: partial-thickness tear
RCT: rotator cuff tendinopathy
SAD: subacromial decompression
TNF-α: tumour necrosis factor alpha
