# Supplementary material for: Differences in glutamate receptors and inflammatory cell numbers are associated with the resolution of pain in human rotator cuff tendinopathy
Source: Arthritis Res Ther. 2015 Jul 10;17(1):176. doi: 10.1186/s13075-015-0691-5 (PMC4498529; doi:10.1186/s13075-015-0691-5)
Supplement: Additional file 1: — ‘Appendices’ and consists of Appendix 1, 2 and 3. [file 13075_2015_691_MOESM1_ESM.pdf]

Appendix 1 - Antibodies and staining protocols

Antibody	Raised in	Dilution	Make		Concentration
CD34	mouse	1/50	Dako	M7165	12mg/L
CD45	mouse	1/50	Dako	M0701	-
CD206	Mouse	1/200	Abcam	Ab117644	0.5mg/ml
Glutamate	rabbit	1/100	Millipore	AB5018	-
HIF1α	mouse	1/250	R&D	MAB1935	0.5mg/ml
KA1	rabbit	1/4000	Abcam	Ab67402	1mg/ml
LDH	rabbit	1/250	Abcam	Ab134187	-
mGluR1	mouse	1/250	R&D	MAB4836	0.5mg/ml
mGluR2	rabbit	1/350	abcam	Ab118447	1mg/ml
mGlur7	rabbit	1/150	Abcam	Ab53705	1mg/ml
NMDAR1	mouse	1/140	BD	556308	0.5mg/ml
PCNA	rabbit	1/200	DAKO	M0879	327mg/L
PGP9.5	rabbit	1/200	Thermo	PA1-21097	0.2mg/ml
VEGF	goat	1/200	R&D	AB-293-NA	1mg/ml
Immunoglobulins	rabbit	1/2000	Dako	X0903	-
IgG1	mouse	1/600	Dako	X0931	-
Immunoglobulins	goat	1/40	Dako	F0250	-

A1.1 Deparaffinisation and target retrieval

1. Working solution prepared according to manufacturer's instructions: 30mls of EnVision ™ Flex high pH target retrieval solution mixed with 1470mls of deionised water to make up 1.5l of solution.
2. PT Link Tank filled with 1.5l of working solution in order to cover tissue sections.
3. PT Link set to preheat solution to 65°C.
4. Formalin fixed paraffin-embedded tissue sections immersed into pre-heated target retrieval solution and incubated at 97°C for 20 minutes.
5. Sections left to cool to 65°C in PT Link.
6. Slide racks removed from PT Link and immersed in PT Link Rinse Station containing diluted, room temperature Dako Wash Buffer (20X).
7. Slides left in the Wash Buffer for 5-10 minutes.

A1.2 Haemotoxylin and Eosin staining protocol

The programming steps and Incubation times are pre-programmed on the DAKO® Autostainer as follows:

1. Modified Mayer's Hematoxylin – 10 minutes
2. Double Rinse
3. Bluing Reagent – 2 minutes
4. Rinse + Blow
5. Eosin Y – 1 minute
6. Double Rinse
7. 100% ethanol – 1 minute
8. Blow

Use a minimum of 400 µL of reagent per slide. Prior to starting a run, slides are deparaffinised and rinsed in water. The slides are pre-soaked in a working solution of DAKO® Special Stains Wash Buffer for 5 minutes. The slides are then placed on the

DAKO® Autostainer and the run is begun. After the run is complete, the slides are dehydrated through 2 changes of 100% alcohol and cleared in 2 changes of xylene. Finally the slides are coverslipped with permanent mounting media.

A1.3 Immunostaining protocol without linker for mouse/rabbit raised primary antibodies (IS-1)

1.	Rinse	1 minute	Buffer
2.	Peroxidase Block	5 minutes	200µl EnVision FLEX Peroxidase Blocking Solution
3.	Rinse	1 minute	Buffer
4.	Primary antibody	Variable time	200µl primary antibody raised in mouse or rabbit
5.	Rinse	1 minute	Buffer
6.	Detection	20 minutes	200µl EnVision FLEX/HRP
7.	Rinse	1 minute	Buffer
8.	Rinse	1 minute	Buffer
9.	Stain	5 minutes	200µl DAB substrate working solution
10.	Stain	5 minutes	200µl DAB substrate working solution
11.	Rinse	1 minute	Buffer
12.	Counterstain	5 minutes	200µl EnVision FLEX Haemotoxylin
13.	Rinse	1 minute	Deionised water
14.	Rinse	5 minutes	Buffer
15.	Rinse	1 minute	Deionised water

A1.4 Immunostaining protocol without linker for goat/sheep raised primary antibodies (IS-2)

1.	Rinse	1 minute	Buffer
2.	Peroxidase Block	5 minutes	200µl EnVision FLEX Peroxidase Blocking Solution
3.	Rinse	1 minute	Buffer
4.	Primary antibody	Variable time	200µl primary antibody raised in sheep or goat
5.	Rinse	1 minute	Buffer
6.	Secondary antibody	20 minutes	200µl Anti Goat or Sheep Ig HRP
7.	Rinse	1 minute	Buffer
8.	Detection	20 minutes	200µl EnVision FLEX/HRP
9.	Rinse	1 minute	Buffer
10.	Rinse	1 minute	Buffer
11.	Stain	5 minutes	200µl DAB substrate working solution
12.	Stain	5 minutes	200µl DAB substrate working solution
13.	Rinse	1 minute	Buffer
14.	Counterstain	5 minutes	200µl EnVision FLEX Haemotoxylin
15.	Rinse	1 minute	Deionised water
16.	Rinse	5 minutes	Buffer
17.	Rinse	1 minute	Deionised water

A1.5 Immunostaining protocol with linker for mouse/rabbit raised primary antibodies (IS-3)

1.	Rinse	1 minute	Buffer
2.	Peroxidase Block	5 minutes	200µl EnVision FLEX Peroxidase Blocking Solution
3.	Rinse	1 minute	Buffer
4.	Primary antibody	Variable time	200µl primary antibody raised in mouse or rabbit
5.	Rinse	1 minute	Buffer
6.	Linker	15 minutes	200µl EnVision FLEX+ Rabbit or Mouse Linker
7.	Rinse	1 minute	Buffer
8.	Detection	20 minutes	200µl EnVision FLEX/HRP
9.	Rinse	1 minute	Buffer
10.	Rinse	1 minute	Buffer
11.	Stain	5 minutes	200µl DAB substrate working solution
12.	Stain	5 minutes	200µl DAB substrate working solution
13.	Rinse	1 minute	Buffer
14.	Counterstain	5 minutes	200µl EnVision FLEX Haemotoxylin
15.	Rinse	1 minute	Deionised water
16.	Rinse	5 minutes	Buffer
17.	Rinse	1 minute	Deionised water

A1.6 Immunostaining protocol with linker for goat/sheep raised primary antibodies (IS-4)

1.	Rinse	1 minute	Buffer
2.	Peroxidase Block	5 minutes	200µl EnVision FLEX Peroxidase Blocking Solution
3.	Rinse	1 minute	Buffer
4.	Primary antibody	Variable time	200µl primary antibody raised in sheep or goat
5.	Rinse	1 minute	Buffer
6.	Secondary antibody	20 minutes	200µl Anti Goat or Sheep Ig HRP
7.	Rinse	1 minute	Buffer
8.	Rinse	1 minute	Buffer
9.	Linker	15 minutes	200µl EnVision FLEX+ Rabbit Linker
10.	Rinse	1 minute	Buffer
11.	Detection	20 minutes	200µl EnVision FLEX/HRP
12.	Rinse	1 minute	Buffer
13.	Rinse	1 minute	Buffer
14.	Stain	5 minutes	200µl DAB substrate working solution
15.	Stain	5 minutes	200µl DAB substrate working solution
16.	Rinse	1 minute	Buffer
17.	Counterstain	5 minutes	200µl EnVision FLEX Haemotoxylin
18.	Rinse	1 minute	Deionised water
19.	Rinse	5 minutes	Buffer
20.	Rinse	1 minute	Deionised water

A1.7 Dehydration, clearing and permanent mounting

1. Slides removed from Autostainer slide racks and placed into Leica slide racks.
2. Dehydrate 1 minute 70% alcohol
   1 minute 90% alcohol
   1 minute 100% alcohol
3. Clear 5 minutes Xylene

   5 minutes Xylene

4. Mount Pertex mountant and application of cover slips.

Appendix 2 – modified Bonar scoring system

Modified Bonar Score for the rotator cuff	Grade 0	Grade 1	Grade 2	Grade 3
Cell morphology (four fields of view 100X)	Inconspicuous elongated spindle shaped nuclei with no obvious cytoplasm	Increased roundness: nucleus becomes more ovoid to round in shape without conspicuous cytoplasm	Increased roundness and size: the nucleus is round, slightly enlarged and a small amount of cytoplasm is visible	Nucleus is round. , large with abundant cytoplasm and lacuna formation (chondroid change)
Collagen arrangement (one field of view 100X)	Collagen arranged in tightly cohesive well demarcated bundles with a smooth dense bright homogenous polarization pattern with normal crimping	Diminished fibre polarization; separation of individual fibre bundles but with maintenance of overall bundle architecture Non homogeneous polarization	Bundle changes; separation and loss of demarcation of fibre bundles, giving rise to expansion of the tissue overall and clear loss of normal polarization pattern	Marked separation of fibre bundles with complete loss of architecture
Cellularity (mean and standard deviation of 100X images of whole section)	Mean cell count <12 and standard deviation of count <7	Either mean ≥ 12 and < 16, and/or standard deviation of count ≥ 7 and < 10	Either mean ≥16 and < 20, and/or standard deviation of count ≥10 and < 13	Either mean ≥ 20, and/or standard deviation ≥ 13
Vascularity (mean vessel count of 100X images of whole substance)	>1.2	< 1.2 and ≥ 0.8	< 0.8 and ≥ 0.4	< 0.4
Ground substance (one field of view 100X)	Not stainable ground substance	Stainable mucin between bundles but bundles still discrete	Stainable mucin within bundles with loss of clear demarcation of bundles	Abundant mucin throughout the section with inconspicuous collagen staining

Appendix 3 – Primer details

Primer	Company		5' to 3' sequence<br>(or Qiagen code if QuantiTect)	Annealing temperature (°C)	Amplicon size (bp)
Beta actin	Eurofins	Fwd<br>Rev	CATGTACGTTGCTATCCAGGC<br>CTCCTTAATGTCACGCACGAT	59	
CASP3	Qiagen		QT00023947		147
COL1A1	Qiagen		QT00037793		118
COL3A1	Qiagen		QT00058233		95
GAPDH	Qiagen		QT00079247		95
GRIK1	Primer Design	Fwd<br>Rev	CCTCTCTCCTTCTTCGTTAGTCT<br>TCTCCCTCATTCGTCCTTTGG	57	99
GRIN1	Primer Design	Fwd<br>Rev	GCCATCCTAGTTAGCCATCCA<br>ACGGGTATGCGGTAGAAGC	57	89
GRM1	Primer Design	Fwd<br>Rev	GCTGGCATCTTCCTTGGTTATG<br>GTCACTAAAGCAGAGTAGCACATC	58	125
GRM2	Qiagen		QT00020356		133
GRM5	Primer Design	Fwd<br>Rev	GTGACCCTGAACCCATTGC<br>ATGATGAAGACTACAGTAACAAAACAG	56	82
GRM7	Qiagen		QT00069727		133
IL1B	Qiagen		QT00021385		117
LDHA	Primer Design	Fwd<br>Rev	GGAATGAATGTTGCTGGTGTCT<br>GGATGTGTAGCCTTTGAGTTTGA	57	141
MRC1	Primer Design	Fwd<br>Rev	TGGGTTCCTCTCTGGTTTCC<br>CAACATTTCTGAACAATCCTATCCA	56	114
mTOR	Primer Design	Fwd<br>Rev	CTGATGCTGGACCGTCTGA<br>TCTTGTTAGTCTAAATGGAATCTTCTC	56	114
PCNA	Primer Design	Fwd<br>Rev	GCCGAAACCAGCTAGACTTT<br>TCGTTGATGAGGTCCTTGAGT	57	140
TBP1	Qiagen		QT00000721		132
TNF1	Qiagen		QT00029162		98
UCLH1	Qiagen		QT00092666		110
VEGF	Primer Design	Fwd<br>Rev	CCAGGAAAGACTGATACAGAACG<br>GGTTTCTGGATTAAGGACTGTTC	57	93

Appendix 4 – P values sorted according to the Benjamini-Hochberg procedure

Sorted P values	d x i/n	Is P ≤ d x i/n?	Tissue marker
0.0004	0.003	yes	CD206
0.0019	0.006	yes	mGluR7
0.0028	0.009	yes	KA1
0.004	0.013	yes	VEGF
0.007	0.016	yes	LDH
0.0079	0.019	yes	PGP9.5
0.038	0.022	no	CD45
